# Patterns of correlation of facial shape with physiological measurements are more integrated than patterns of correlation with ratings

**DOI:** 10.1038/srep45340

**Published:** 2017-03-28

**Authors:** S. Windhager, F. L. Bookstein, E. Millesi, B. Wallner, K. Schaefer

**Affiliations:** 1 Department of Anthropology, University of Vienna, Althanstrasse 14, 1090 Wien, Austria; 2 Department of Theoretical Biology, University of Vienna, Althanstrasse 14, 1090 Wien, Austria; 3 Department of Statistics, University of Washington, Box 354322, Seattle, WA 98195-4322, USA; 4 Department of Behavioural Biology, University of Vienna, Althanstrasse 14, 1090 Wien, Austria

## Abstract

This article exploits a method recently incorporated in the geometric morphometric toolkit that complements previous approaches to quantifying the facial features associated with specific body characteristics and trait attribution during social perception. The new method differentiates more globally encoded from more locally encoded information by a summary *scaling dimension* that is estimated by fitting a line to the plot of log bending energy against log variance explained, partial warp by partial warp, for some sample of varying shapes. In the present context these variances come from the regressions of shape on some exogenous cause or effect of form. We work an example involving data from male faces. Here the regression slopes are steepest, and the sums of explained variances over the uniform component, partial warp 1 and partial warp 2 are greatest, for the conventional body mass index, followed by cortisol and, lastly, perceived health. This suggests that physiological characteristics may be represented at larger scale (global patterns), whereas cues in perception are of smaller scale (local patterns). Such a polarity within psychomorphospace, the global versus the focal, now has a metric by which patterns of morphology can be modeled in both biological and psychological studies.

“Human facial diversity is substantial, complex, and largely scientifically unexplained”^1^. The human face is an important source of information for social interactions and for scientists alike. A face advertises, among other things, a person’s sex, age, hormonal status, previous environmental exposure, health, interpersonal attitudes, and emotions. The study of faces and what they communicate in this way integrates genomics, human behavioral biology and life history, evolutionary psychology, and biological anthropology. Ultimately the theory of these relationships is an evolutionary one: that the human body and face have been shaped by selective forces throughout our evolutionary history in response to natural and social environments. Facial morphology thereby occupies the middle of a causal chain whereby biological factors such as age, sex, and body composition are reflected in facial and bodily characteristics that then serve as cues in person perception and the consequent behaviors.

Correlational studies have identified some links between physical characteristics and social inference, but usually fail to identify the specific morphological pathways underlying the inferences. Morphometric face analysis, however, has demonstrated that quantification of the morphological cues is crucial^2^,^3^,^4^. For studying correlates of facial shape variation, researchers are now turning to geometric morphometric (GMM) methods, which can combine biological factors, shape information, and trait inference in the same data space. In 2005, Schaefer and colleagues were the first to make use of this possibility in the analysis of faces^5^,^6^,^7^. In Schaefer *et al*. 2009, the approach was made explicit in a review article and given the name “psychomorphospace”^8^. Since then GMM has been applied in face research by several research groups, e.g., refs 9, 10, 11, 12.

Instead of using distances, angles, or ratios, GMM is based on a complete multivariate analysis of the locations (that is, the Cartesian coordinates) of a designed set of landmark and semilandmark points taken all together. The most important advantage of GMM is that it preserves the relative spatial relationships of the landmarks and semilandmarks throughout the analysis. The first step is standardizing for position, size and orientation of the faces using a least squares criterion (Procrustes distance). Thereafter, linear regressions of the shape coordinates on the variable of interest quantify and depict the association of this variable with facial shape. This paper exploits a GMM technique only a couple of years old that decomposes the result of such a shape regression into variation at both large and small scales, in order to localize and visualize the relative predominance of its large-scale versus small-scale features. The publication introducing this method^13^ focused on providing modern paleobiology with a tool to differentiate among integration, dis-integration, and self-similarity. But the concepts and equations entailed directly transfer to the fundamental questions in face research. Integration implies a large contribution from large-scale variation (global patterns), whereas dis-integration can be interpreted as a higher amplitude of small-scale variation (local patterns) in facial signals. Thus, this morphometric notion of integration is based as much in the geometry of landmark placement as in their correlations^13^. Even though small-scale features may be correlated, they cannot be “integrated” in our morphometric sense unless the deformations of the spaces around them are correlated as well, meaning the integration must be at large scale.

Our article exemplifies the new analysis using regressions of male faces on three traits typically associated with facial shape: a physical trait (body mass index, BMI), an endocrinological measure (salivary cortisol), and a rating (perceived health). This short list is not intended as an exhaustive directory of traits, only as a preliminary survey of the range of scaling dimensions that might be exploited in today’s range of studies of facial shape and trait attribution.

We are learning steadily more about the facial correlates of body composition and endocrinological status, on the one hand, and ascribed personal characteristics in social perception, on the other. Studies of facial masculinity or femininity and facial attractiveness have a longer history, while the topics of facial cues to body mass index or health are receiving increasing attention. Current studies in these areas exploit a variety of methods, including linear distances and angles, techniques of computer vision, and geometric morphometrics. Yet one cannot say whether it is the individual features (eyebrows, eyes, nose, mouth – all aspects of local variation) or instead general aspects of shape such as the overall shape of the facial outline or facial width-to-height ratio (global variation) that carry most of the signal. Early approaches to this puzzle included the dissection of the face into single features and their isolation and systematic variation via line drawings or identi-kits (e.g., ref. 14), along with single- and multiple-feature variation^15^. Although these approaches proved productive, information about natural variation and covariation of the features could not be included and, in spite of modern software and feature manipulations (e.g., refs 16,17) cannot be retrieved. By distinguishing between local and global variation, the GMM approach presented in this paper supersedes such testing of isolated single features.

It is difficult to derive hypotheses about the relative contributions of large- and small-scale variation from the existing literature. Since faces are biological systems operating under functional constraints, a certain degree of integration is to be expected. This expectation is consistent with the observation that so far no biological data set was analyzed in which the slope of the regressions we will be highlighting was closer to zero (indicating no integration) than −0.56^13^. One also might expect that facial shape changes consequent to biological processes (body fat storage, water retention) would be more global than psychological signals would be. That is, even given the compartmentalization of fat or extracellular fluids (e.g., ref. 18), these are likely to be more uniformly distributed around the face than are social signals. For example, as Keating^15^, p. 68, concludes, “variations in eye size or lip thickness alone [are the] reliable dominance cues.” Another hint that local effects dominate social perception is the finding that even neutral facial expressions convey emotional meaning because certain purely histological traits, such as downturned corners of the mouth due to fatty pads or water retention, mimic emotional expressions^19^, while certain ambiguous emotional displays, such as lowering the eyebrows and upturning the corners of the mouth, bear emotional valence (e.g., refs 20,21). Highly transient states like these that owe to single muscle units likely represent the most dis-integrated facial features. Other forms of social inference might be intermediate, relying on both local and global features. Taken as a whole, our new approach may not rewrite these intuitive understandings, but it will quantify them for the first time.

## Material and Methods

### Participants

Frontal photographs (procedure below), body height via anthropometer, body composition (Tanita TBF 300), and saliva samples (see below) were collected from 34 ethnically Central European men from the Viennese student population. They were recruited at the Centre for Organismal Systems Biology of the University of Vienna. Each participant was informed about the measurement procedure, subsequent data use, and the right to withdraw from the study at any time; all gave their informed written consent. All protocols were in accordance with the Declaration of Helsinki.

Subjects’ age ranged from 19 to 27 years; body mass index [body weight (kg)/body height (m)^2^; BMI], from 17.7 to 34.9 (22.9 ± 4.1). BMI and body fat proportion were highly correlated (*r*_*s*_ = 0.881); we chose to present BMI because it is the more common choice of previous researchers into facial adiposity.

### Hormone sampling

Each participant provided six saliva samples, three per session at intervals of about 20 minutes. Each session started between 08:00 and 09:30 a.m. Participants were advised not to eat or drink for at least one hour before the data collection, not to brush their teeth that morning (in order to avoid the risk of small bleeds), and not to be involved in sports or sexual activities, to drink alcohol or caffeine, or to take drugs over the 12 hours preceding the measurement session. Salivary samples were frozen at −20 °C and analyzed jointly in the endocrine lab at the Department of Behavioural Biology of the University of Vienna. Cortisol concentration was quantified by a microtiter plate enzyme immunoassay (EIA) using procedures developed by Palme and Möstl^22^. Repeated measurements of duplicate pool samples revealed a mean inter-assay coefficient of variation of 11.6%; the mean intra-assay coefficient of variation was 14.5%, which is the usual variation for analyses using group-specific enzyme immuno-assays^23^,^24^. Individual samples with high discrepancies between duplicate samples were excluded before averaging. Mean cortisol values per subject ranged between 14.8 and 52.3 ng/ml (29.9 ± 8.9 ng/ml).

### Rating study

**Figure 1:**
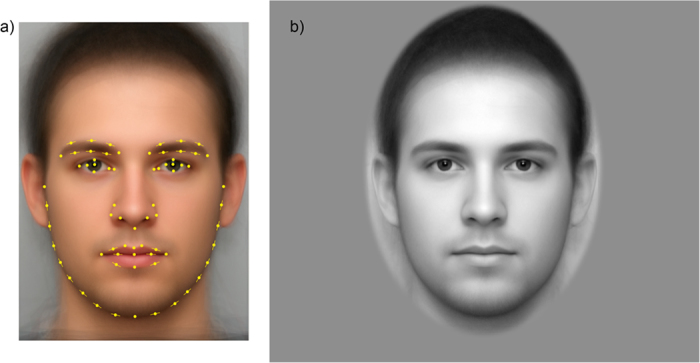
(**a**) Landmark scheme. Thirty-seven point landmarks and thirty-four semilandmarks (–) were digitized on each facial portrait. Subsequently, their *x*- and *y*-coordinates were subjected to a generalized Procrustes superimposition with additional steps for sliding and symmetrization^26^. (**b**) Greyscaled version of the same portrait on standardized background as vignetted by a blurred ellipse. The face in this figure is the actual average of all the sample faces after each was unwarped to the sample average configuration.

Each rater (39 male, 62 female; ethnically Central European; 20–45 years, 33 ± 6.7 years) rated each of the 34 male faces (grey-scaled and masked by a blurred ellipse, Fig. 1) in pseudo-random order on a computer screen using sliders with a hidden range from 0 (unwell) to 100 (healthy appearance). Participation in the rating study was wholly voluntary; all participants completed the procedure. To account for individual variation in rating ranges, data were rank-ordered within each rater; then the median of the 101 scores for each photograph was taken as the measure for perceived health for the corresponding photograph. Median ranks ranged from 5 to 26.75 (17.2 ± 5.9) out of 34, a gratifyingly wide span that sustained the further analysis we are about to report.

### Facial photographs and landmark data

Frontal photographs were taken at 350 cm with the head adjusted according to the Frankfort horizontal and a neutral facial expression. We used a digital reflex camera (Canon EOS 40D) with a 200 mm lens positioned at eye height.

A total of 71 landmarks and semilandmarks were digitized to capture facial shape (Fig. 1). Landmark definitions basically match the earlier operationalization of Windhager and colleagues^25^.

### Shape analysis

**Figure 2:**
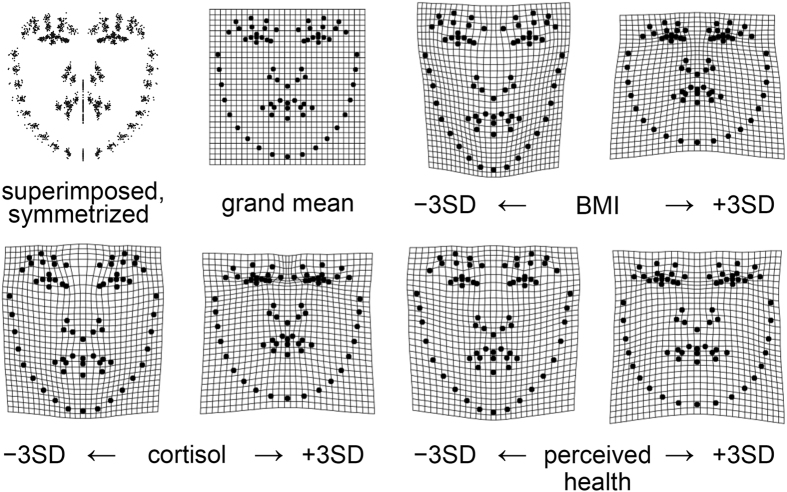
Visualization of symmetrized shape regressions upon BMI, cortisol and health rating by thin-plate spline (TPS) deformation grids. The average landmark configuration corresponds to the undeformed grid. The complete symmetrized scatter of all shape coordinates that generate the grand mean is presented to its left. The deformations correspond to a decrease (left) or an increase (right) of 3 standard deviations of the predictor variable: BMI, top right pair; cortisol, bottom left pair; health rating, bottom right pair.

The initial steps in the present research dataflow were those that have become standard in Procrustes studies of facial form^8^,^27^. Landmark and semilandmark locations from Fig. 1, after Procrustes superimposition, were regressed on correlates of facial form of three different types: physiological measurements (here, BMI), hormonal transients (here, salivary cortisol), and perceptions by others (here, a health rating). The regression vectors that prove conventionally significant by the usual permutation tests may be visualized as thin-plate spline grid deformations from the mean form to the predicted forms that lay three standard deviations from the mean in either direction (Fig. 2).

At this point we invoked the novel procedure just introduced to the community of disciplines concerned with evolution: the formal construction of a dimension of spatial scaling corresponding to any shape phenomenon of interest (here, any of these regressions). For a detailed mathematical explanation of this procedure, see ref. 13. The approach is an extension to our shape morphometrics of a formalism already somewhat familiar from studies of Brownian motion. Mandelbrot’s notion of fractal dimension^28^ is based on early work by Perrin^29^ and others confirming Einstein’s self-similar model of diffusion. In Brownian motion, as observed in the laboratory, the statistical properties of any segment of the process are independent of the duration of that segment except for one single parameter, the diffusion coefficient (or, for a random walk, the step variance). A diffusion four times as long as another looks exactly the same except for a scaling of amplitude by a factor of 2 (the square root of 4).

Bookstein^13^ shows how this same notion of scaling can be converted from time comparisons to space comparisons by use of the machinery of principal and partial warps that is already part of the standard thin-plate-spline morphometric toolkit^27^. This machinery has been part of GMM since the beginning (cf. refs 26, 27 and 30), but these tools are not applied as often as the other parts of this useful technological praxis for shape analysis. Briefly, any individual shape of some landmark configuration (here, the shape of a face) can be represented as the deformation of the sample average shape. The thin-plate spline diagram that GMM typically uses to convey one of these deformations has a specific *bending energy,* a net quantity of what would be actual physical energy if the situation were that of a metal plate bending perpendicular to the picture plane. Bending energy turns out to be a quadratic form (in effect, a sum of squares) in the coordinates of the landmark points themselves. And, just as sines and cosines are a conveniently simple representation of the way a musical sound can be expressed in terms of pure tones, so the principal warps are a conveniently simple representation of the ways that any single shape change can be re-expressed as a superposition of these rhetorically useful forms of “pure bending at some particular scale.” Principal warps are geometrically orthogonal components corresponding to deformations at different geometric scales (analogous to different powers in polynomial curve fitting^31^). A partial warp is just the combination of two copies of the same principal warp, once for the horizontal coordinate of a facial shape, once for the vertical coordinate. Finally, the uniform component is the part of the change that comes from patterns that are free of bending – the so-called *affine transformations* that leave parallel straight lines parallel.

Once each observed shape is represented in terms of this new set of descriptors, the uniform component together with all the partial warps, the analysis of integration just introduced into the evolutionary literature^13^ is launched, as follows. One begins by removing all the shape variance that corresponds to the uniform shape changes (here, changes in height/width ratio of these faces). In a context of two-dimensional data (such as our facial photos), if the variance of every partial warp is exactly proportional to the reciprocal of its bending energy, then the nonuniform shape variance of every small square of landmarks and semilandmarks, regardless of size, position, or orientation, will be the same. If the variance of partial warps drops faster than their bending energy rises, the transformation can be said to be more integrated, with greater variability at the larger scales of shape features. Conversely, if partial warp variance drops more slowly than bending energy rises, the transformation is more dis-integrated, with more variability of the smaller-scale structures than would be predicted by the large-scale variation. (Our standard Procrustes null distribution lies in an extreme position on this scale, with the variance of every partial warp exactly the same a priori. This is one reason it is an inadvisable choice for applications in biological morphometrics^32^).

One gets from a landmark-based data representation to an estimate of this scaling dimension for any particular shape phenomenon by carrying out one additional regression (see examples in the penultimate figure below). The new regression fits a line to a plot of log bending energy against log partial warp variance for all the partial warps representing the transformation under study. In plots like this one, the first partial warp is the pattern of nonuniform shape change with the least bending per unit Procrustes length–this is usually a bending of the long axis of the form under study, and can be in the x-direction, the y-direction, or any combination. The second partial warp typically complements the first one by some version of a cubic (S-shaped) bend, likewise in any combination of x- and y-directions, and so on until the last partial warp, which is usually the relative displacement of the pair of landmarks at closest spacing to one another. Whatever the reference form, the partial warps provide an ordination of all its possible shape changes along the single dimension of steadily greater and greater bending per unit Procrustes length (of the deformation). The fitted regression slope is a summary measure of the steepness of fall of this ordination. Slopes steeper than −1 correspond to integrated processes (such as growth) that affect all regions of the form by a small number of quite powerful 1-factors. Slopes shallower than −1 represent patterns of the opposite connotation, patterns that are much less correlated from locus to locus across the form. In-between are the strictly self-similar processes, of slope exactly −1. These are the analogues of Brownian motion for this domain of shape features—transformations that have the same nonuniform variance (transformation of squares into trapezoids or kites), as a proportion of starting scale, regardless of that scale.

Of the regressions of form on its correlates that are considered in this paper, one is an integrated pattern, one is a dis-integrated pattern, and one is a self-similar pattern. We show the regressions of variance on bending energy responsible for this classification and, back on the picture of the face, the evident variations of predicted landmark shifts that correspond to this taxonomy of scaling regimes. We also interpret the difference, which is substantial, in terms of the different origins of these shape regressions in development versus perceptual processes.

We used F. James Rohlf’s computer programs tpsUtil and tpsDig2 for landmark digitization, tpsRelw for sliding of the semilandmarks, tpsRegr for the shape regressions, and tpsSuper for the image unwarping and averaging^33^. The analysis of spatial scaling was carried out in S-Plus.

## Results

The principal scalar measurements of this study were nearly uncorrelated among themselves. Rank-correlations were as follows: BMI and cortisol, 0.089; BMI and health rating, 0.157; cortisol and health rating, 0.279 (*n.s*. for our *N* = 34). Facial shape variation was strongly predicted by each of BMI, cortisol, and health rating separately. Each shape regression was significant (all *p*’s ≤ 0.05 over 1000 permutations). With symmetrized faces, the fraction of variance explained by BMI was 18.5%, by cortisol, 10.7%, and by health rating, 6.0%.

**Figure 3:**
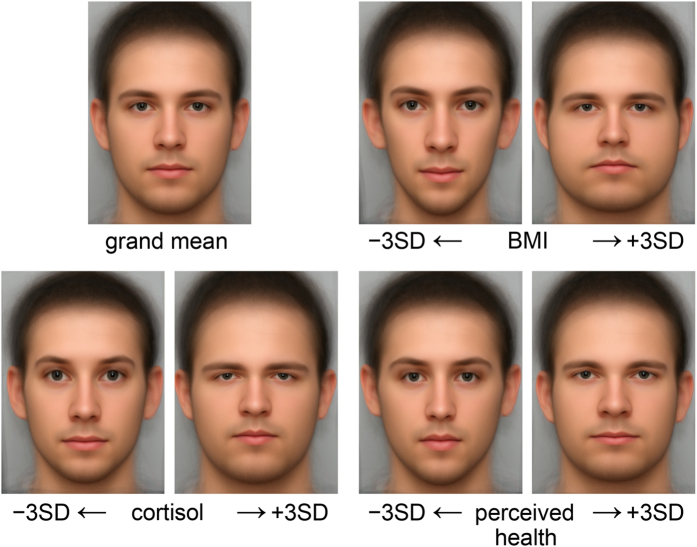
Computed morphs of the averaged unwarped image (GM morphs) depicting the same shape regressions and configurations as the thin-plate splines (Fig. 2): the sample average as well as the facial shapes corresponding to low (minus three standard deviations) and high (plus three standard deviations) of BMI, cortisol, and health rating.

Before proceeding to the detailed spatial analysis, we guide the reader through the typical verbal interpretation of opposite pairs of grids (Fig. 2) and averaged unwarped images (GM morphs, Fig. 3). The male facial shape associated with low BMI in our data is mainly characterized by an elongated facial outline with the sensory organs comparatively larger and more widely spread out over the area of the face. This general pattern is emphasized by higher eyebrows, a relatively larger visible part of the sclera and iris, a longer nose, fuller lips, upturned corners of the mouth, and a more pointed chin. In contrast, men with a high BMI tend to have a rounder face with more centrally situated and comparatively smaller sensory organs. Likewise, the sclera and the iris are less visible, the mouth has more downturned corners, and the chin appears to be wider and rounder. The general facial correlates of low salivary cortisol somewhat resemble those for low BMI except for the shape of the eye region (eyes that are more almond-shaped). In contrast, high salivary cortisol is related to eyes that are more slit-like, with upper lid regions that look almost swollen. Generally, the facial outline widens with increasing salivary cortisol as with increasing BMI. Morphs visualizing the different health ratings hardly differ in overall size and location of the sensory organs in relation to the whole face (as they did for comparisons over the range of BMI or salivary cortisol). Still, the shapes of sensory organs are not the same along the attributed health gradient. For lower health ratings, the eyes are relatively rounder, the nose thinner and longer, and the mouth narrower but framed by thicker lips. In contrast, higher health ratings are characterized by relatively more elliptical eyes with lower and straighter eyebrows, as well as a shorter and wider nose. The lips are relatively thinner and the mouth is wider. The overall face is less oval, but rather more square than one with lower attributed health.

**Figure 4:**
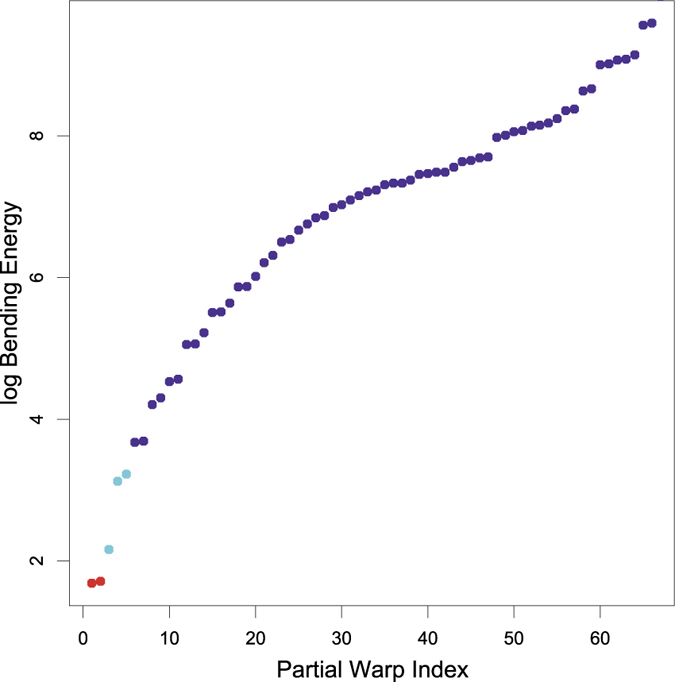
Color key for the shape changes per partial warp depicted in Fig. 5. The first two partial warps (in red) correspond to the non-uniform components with the least bending energy per unit Procrustes length. The subsequent three partial warp contributions are coded in cyan, and the others – representing small-scale, local variations – in purple. Note the log-scale along the vertical axis.

**Figure 5:**
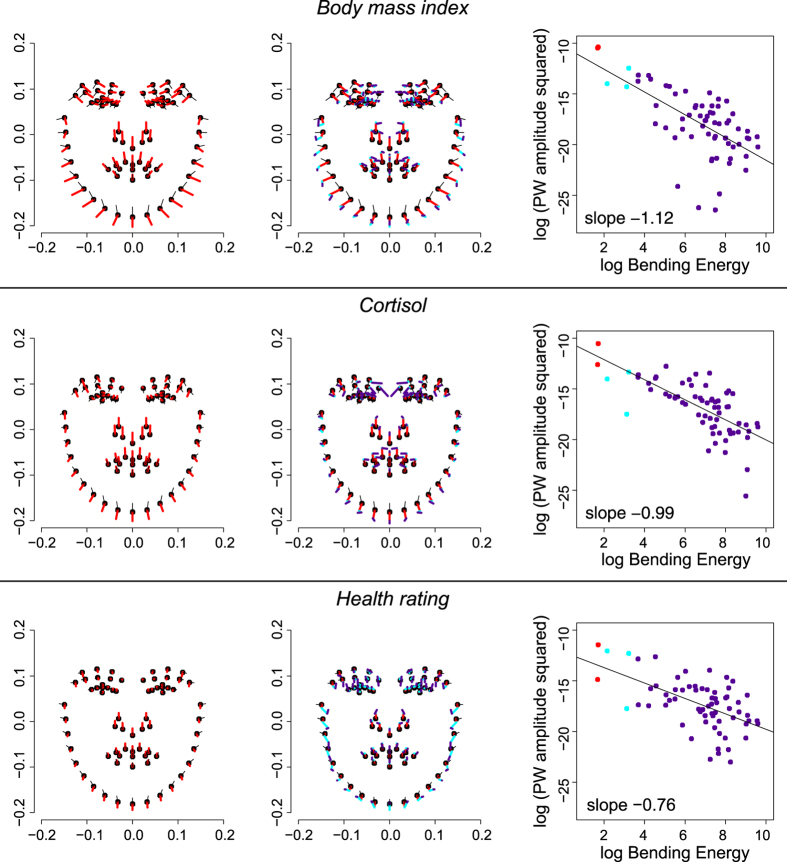
Graphical representation of spatial scaling. The left column depicts the contribution of the uniform component (in black) as well as the vectors for partial warp 1 and partial warp 2 (both in red). This stands for large-scale variation. In the middle column, the other partial warps, representing small-scale variation, are added (the next larger three in cyan, the others in purple). The uniform component together with all partial warps gives the deformations depicted in Fig. 2. The right column gives the log partial warp amplitude squared together with the log bending energy for each partial warp. The frame at upper right is not missing a red dot; rather, there are two red dots, which overlap nearly perfectly on the scale of this vertical axis. Lines are the regressions whose slopes indicate the level of (dis-)integration pattern by pattern.

In order to quantify spatial scaling for each of these regressions, shape variance is first split into uniform and non-uniform shape changes. The relative contribution of the uniform component varied by predictor variable: 31.5% for BMI, 26.8% for cortisol, 25.2% for health rating (Table 1). The uniform component is depicted as black lines in the penultimate figure. Thereafter, the scaling dimension is estimated via the regression of log partial warp variance on log bending energy for all the partial warps of the transformation (Fig. 4; Fig. 5, third column). By definition, the first partial warps have the least bending per unit Procrustes length. Table 1 shows that the sum of variances over the uniform component, partial warp 1 and partial warp 2 (the largest scale contributions) is highest for BMI (86%), followed by cortisol (73%) and health rating (54%). The variance at these highest three scales is 0.95 × 10^−4^ for BMI, 0.49 × 10^−4^ for cortisol, and 0.24 × 10^−4^ for health rating.

For BMI, the partial warp variance drops faster than the bending energy rises (Fig. 5, third column). The corresponding slope of −1.12 stands for an integrated pattern. Cortisol shows an intermediate pattern between integration and dis-integration with a slope of −0.99 (which Bookstein characterized as “self-similarity”^13^). Although there is a major effect of components at larger scale, there are substantial local effects as well (Fig. 5, second row, middle column). These are found mainly in the eye region, the relative distance between the nose and the mouth, and the chin. In interpreting these displacement diagrams, the reader should emphasize the visual extent of the colors per se – the net lengths of our three selected subdomains of partial warps, irrespective of their direction – and should not be concerned with the appearance of “outliers,” as the partial warps are precomputed patterns correlated over all 71 of the (semi-)landmarks of the design. The visual impact of the red segments decreases down the figure, while those of the cyan and purple segments increase. The most dis-integrated pattern was found for the health rating. This means that the corresponding shape changes are much less correlated from locus to locus across the male face. The slope of −0.76 here is significantly shallower than the regression slope of −1.12 for BMI (*p* = 0.002). Small-scale effects predominate (Fig. 5, bottom row).

## Discussion

**Figure 6:**
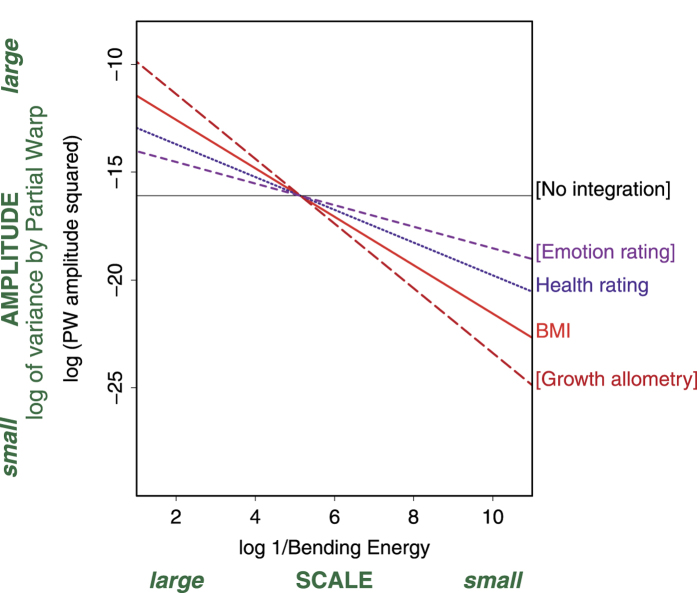
Spatial wavelengths: the “colors” of the face as viewed frontally. This schema visualizes the different structures of correlation across shape regressions by dissecting the scale of variation involved. While biological variables are encoded in rather large scale (global patterns), perceptual outcomes tend to be of smaller scale (local patterns). Brackets indicate hypothetical scenarios.

It has become customary to analyze correlates of facial form by regressing Procrustes shape representations of that variation on their hypothesized causes or effects. Both the causes of that variation (here, the BMI index) and the effects of that variation (here, perceived “health”) can be detected and described by strong regressions of this sort. Our results have shown how regressions like these can sometimes be differentiated by their apparent geometric scale. We might summarize the findings and their interpretation in the form of the oversimplified diagram in Fig. 6. Physiological effects upon form appear more integrated than hormonal correlates of form, which are, in turn, more integrated than the apparently multifocal perceptual effects of form that our brains invoke implicitly in the course of rating studies. The finding suggests a polarity within psychomorphospace studies: contrasting global versus focal patterns of morphology.

Figure 6 incorporates a subtle color-coding. You are familiar with color as wavelengths of light: red has the longest wavelength in the visible spectrum, blue the shortest. Figure 6 exploits this color spectrum by “coloring” the wavelengths of bending as if all these regressors, causes and ratings alike, were filters on the same unchanging data set of shapes. The diagram copies the top right and bottom right regression lines from Fig. 5, “BMI” and “Health rating”, and adds three others corresponding to hypothetical processes that go beyond the data of the present paper. All these lines are to the same axes as in the right column of Fig. 5. The line labeled “Growth allometry” has slope -1.5, the estimated slope for allometry from an analysis of growing rodent skulls^13^. The line labeled “Emotion rating” expresses the conjecture that a rating of an emotional state, such as anger (think of the role of the eyebrows in conventional cartoon renderings of this emotion), will focus even more sharply on local features and less on global gradients at the largest scales. Finally, the line labeled “no integration” is the biologically impossible situation modeled by the Mardia-Dryden distribution^26^ where all landmarks vary independently by the same circular Gaussian. In terms of the more conventional language of filtering, we color “BMI” in red because in comparison with selfsimilarity it is like a red filter, emphasizing long spatial wavelengths, and the Health rating is drawn in blue because, like a blue filter, it comparatively emphasizes shorter wavelengths – the partial warps of higher energy at the right on the horizontal scale. Growth allometry should be drawn in the “infrared” on this diagram, even stronger at larger scales and weaker at small scales. Conversely, emotional ratings should be drawn in ultraviolet, with even more amplitude than the health rating at the smaller scales. Finally, the parody of “no integration” is shown in black, as it is incompatible with life. In the metaphor of the filter, this distribution lets no meaningful biological signal through at all.

The sort of large-scale variation represented by the red line in Fig. 6 is conceptually analogous to the *n*-dimensional feature space proposed by Grammer and colleagues^34^, in which correlated features compose a single ornament no matter the spatial extent over which they are correlated. In contrast, single feature approaches such as identi-kits might be regarded as analogous to the small-scale patterns here indicated by the colors of blue or violet.

Certainly, systemic effects are reflected in much more global spatial patterns than single muscle movements are. In this context fat and water distribution seem to be important issues. In young adults (as in our sample), fat in the face looks fairly evenly distributed because of smooth transitions between subcutaneous fat compartments, while ageing leads to abrupt contour changes between these regions^18^. This is the straightforward explanation of why our shape regressions on BMI reveal mainly large-scale changes in facial morphological covariation: all facial regions studied are highly integrated in respect of fat deposit processes. In contrast, increased saliva cortisol concentrations not only influence the overall shape but also have an impact on specific features, in particular on the area around the eyes. Physiologically, circadian secretion rates of cortisol in relation to other hormones or life-style factors can dramatically influence the water exchange between cells and the extracellular space by ion movements along the cell membranes^35^. Also, cortisol administration in healthy men leads to an expansion of extracellular plasma volume^36^. So it would be reasonable to assume that the inter-individual differences in cortisol in our sample might be associated with differences in water retention. And these effects seem quantifiable not only in a more rounded facial outline with somewhat centrally located sensory organs but also locally around the eyes. “Swollen eyes” can safely be added to this phenomenon, since periorbital puffiness is typically caused by fluid buildup around the eyes.

Further differentiation comes from the nature of ratings. Someone who is asked to attribute a certain trait to a face will search for the cues of that trait. In our example, the raters likely pick shape features that in their experience systematically vary with health status. For instance, faces with “apple cheeks” would consistently be assigned a more highly ranked health status than those hollower in the cheek and eye areas (a pattern coherent with our shape estimates for perceived health status, Figs 2 and 3). In our sample of young male faces, health raters might also pay attention to features such as testosterone markers (pointing to a good immune system^37^), body size markers, and physical strength markers, adding up to a “patchwork face” with a masculine and robust appearance (Fig. 3, bottom right morph). For all of these reasons, the spatial scale for a rating is generally less integrated than for a physiological condition. Dis-integration probably peaks for ratings of emotional expressions. We now have a metric to quantify the different spatial scalings of shape changes associated with the various predictor variables.

In our sample, BMI, cortisol, and health rating are hardly correlated at all. In light of this near-independence of causes and effects combined with the visual similarity of some features in the graphs of Fig. 5, we briefly look into the shape regressions themselves and relate our results to other studies on facial correlates of BMI, cortisol, and perceived health.

The facial shape changes associated with increasing BMI parallel those that others have found, e.g., ref. 9. The overall pattern, which seems robust against choice of morphometric method, is predominantly a global effect, an enlarged lower face. In their meta-analysis of two “Caucasian” and two “African” male samples, Coetzee *et al*. note a low but significant positive correlation of BMI with facial width-to-height ratio and a low negative correlation with perimeter-to-area and with cheek-to-jaw-width ratios^38^. Cheek depth and relative lower face width seem to be most affected by nutritional condition (see reference 39 for a review). The pattern in Fig. 5 also closely resembles the deformation induced by rising percentage of body fat in men^25^ and in female adolescents^40^. In a sample of children and adolescents, three normalized distances representing the lower face area were enough to train a machine to predict body weight from facial portraits^41^. Our approach does not require preselection of subsamples or preselection of specific local features as in Henderson and colleagues^42^. When analyzed by shape regressions, such features and contrasts are implicitly embraced by a single pooled analysis of all the landmarks and semilandmarks on all the faces.

This study is one of the first to quantify the facial shape changes that covary with cortisol in young adult males. Moore and colleagues produced composite faces, each an average over five to eight men, to represent the four combinations of high/low cortisol with high/low testosterone^43^. It appears that their results parallel ours: Both of their high cortisol conditions are characterized by a rounded facial outline with eyes, nose, and mouth relatively close together. Due to variation in their head positioning we could not compare aspects of the eyes. Our results also resemble to some extent the ones obtained by Gonzalez-Santoyo and colleagues for young adult women^44^. They averaged ten faces of women with low salivary cortisol concentrations and ten faces of women with high cortisol levels. As for men, more salivary cortisol was associated with a higher facial width-to-height ratio. In contrast, their composite for high cortisol had rounder eyes than the one for low cortisol levels, which is the opposite of the trend that we observed. Our pattern of high cortisol effects is also consistent with the facial appearance of Cushing’s disease (which involves, among other symptoms, chronic overproduction of cortisol). Common signs and symptoms of Cushing’s are a round “moon face” along with weight gain/central obesity, hypertension, thin skin and stretch marks, and muscle weakness^45^,^46^. Cortisol administration in healthy men has been related to an expansion of extracellular plasma volume^36^. Such water retention might also explain the “swollen eyes” or drooping eyelids that we observed as local effect with increasing cortisol concentration.

The percentage of large-scale variation (Table 1) dropped to just over half when the regression was on a rating instead of a physiological measurement. Raters overweighed small-scale variation in face shape when judging the health status of another individual in comparison to global patterns like BMI. One explanation could be that people attend to small-scale variations because of the variety of facial expressions and their importance in interpersonal encounters. Perrett and colleagues (2001, as cited in ref. 47) presented composite images combining the 20% healthiest-looking male students and separately the 20% least healthy-looking. A separate version of their study amplified shape, color and texture differences (all images reprinted in ref. 47). The shape characteristics associated with perceived health paralleled our results. In women, perceived health is associated with upward mouth curvature, but not with eyelid openness^42^.

According to Vernon and colleagues^48^, p. E3353, “despite enormous variation in ambient images of faces, a substantial proportion of the variance in first impressions can be accounted for through linear changes in objectively defined features.’’ We have shown that those “linear changes” arise at a range of geometric scales. It is a logical next step to examine which ratings use which features and how their weights might vary in social perception of other qualities or over the type of person being rated (a child, a woman, a person of a different ethnicity [in which respect see Blais *et al*.^49^ or Tan *et al*.^50^]). In line with the analogy of filtering wavelengths, this approach might ramify into models of neural processing patterns that can then be systematically tested by functional brain imaging or other neurometric laboratory methods.

In conclusion, the methods we presented here augment the information obtained from a shape regression by the patterns of diverse spatial scaling profiles. Not only can we calculate the percentage of variance that is explained by large-scale features, to be compared across predictors and contexts (e.g., biological processes vs. zero-acquaintance guesses), but also the slope of the additional regression serves as a continuous metric, the “color” of the spatial filter. Future studies could extend this paradigm by incorporating ratings of emotions that are presumed to correspond to the least integrated shape patterns. The new method renders the framework for classification of the profiles of psychomorphospace considerably more robust. For example, the dominance of small-scale features in the production of ratings could well help to explain the overgeneralization biases that notoriously afflict rating behaviors in most studies of the particular socially salient ratings that lead to prejudice and ethnic conflicts.

## Additional Information

**How to cite this article:** Windhager, S. *et al*. Patterns of correlation of facial shape with physiological measurements are more integrated than patterns of correlation with ratings. *Sci. Rep.* **7**, 45340; doi: 10.1038/srep45340 (2017).

**Publisher's note:** Springer Nature remains neutral with regard to jurisdictional claims in published maps and institutional affiliations.

## Figures and Tables

**Table 1:** Contribution of large-scale variation as a function of the predictor variable.

Predictor	Total squared length of the regression vector (×10^−4^)^1^	% Uniform component	% PW 1	% PW 2	Cumulative % (Uniform + PW 1 + PW 2)
BMI	1.1078	31.5	26.1	28.4	86.0%
Cortisol	0.6661	26.8	5.1	41.0	72.9%
Health rating	0.4440	25.2	0.8	27.7	53.7%

Total squared lengths of each regression vector together with the relative contributions of the uniform component and the first and second partial warps (PW 1 and PW 2) individually (middle columns) and summed (rightmost column). ^1^This is often called total explained Procrustes variance.

## References

[b1] Claes P. . Modeling 3D facial shape from DNA. PLoS Genet 10, e1004224, doi: 10.1371/journal.pgen.1004224 (2014). 24651127 PMC3961191

[b2] Holland E. Limitations of traditional morphometrics in research on the attractiveness of faces. Psychon Bull Rev 16, 613–615, doi: 10.3758/PBR.16.3.613 (2009). 19451393

[b3] Mitteroecker P., Windhager S., Müller G. B. & Schaefer K. The morphometrics of “masculinity” in human faces. PLoS One 10, e0118374, doi: 10.1371/journal.pone.0118374 (2015). 25671667 PMC4324773

[b4] Todorov A. & Oosterhof N. N. Modeling social perception of faces. Signal Process Mag, IEEE 28, 117–122, doi: 10.1109/MSP.2010.940006 (2011).

[b5] Schaefer K. . Female appearance: facial and bodily attractiveness as shape. Psychol Sci 48, 187–204 (2006).

[b6] Fink B. . Second to fourth digit ratio and face shape. Proc R Soc B 272, 1995–2001, doi: 10.1098/rspb.2005.3179 (2005). PMC1559906 16191608

[b7] Schaefer K., Fink B., Mitteroecker P., Neave N. & Bookstein F. L. Visualizing facial shape regression upon 2nd to 4th digit ratio and testosterone. Coll Antropol 29, 415–419 (2005). 16417137

[b8] Schaefer K., Mitteroecker P., Fink B. & Bookstein F. L. Psychomorphospace—from biology to perception, and back: Towards an integrated quantification of facial form variation. Biol Theory 4, 98–106, doi: 10.1162/biot.2009.4.1.98 (2009).

[b9] Holzleitner I. J. & Perrett D. I. Perception of strength from 3D faces is linked to facial cues of physique. Evol Hum Behav 37, 217–229, doi: 10.1016/j.evolhumbehav.2015.11.004 (2016).

[b10] Kleisner K., Kocnar T., Rubešová A. & Flegr J. Eye color predicts but does not directly influence perceived dominance in men. Pers Individ Dif 49, 59–64, doi: 10.1016/j.paid.2010.03.011 (2010).

[b11] Komori M., Kawamura S. & Ishihara S. Multiple mechanisms in the perception of face gender: Effect of sex-irrelevant features. J Exp Psychol Hum Percept Perform 37, 626–633, doi: 10.1037/a0020369 (2011). 20822297

[b12] Wolffhechel K. . Testing the utility of a data-driven approach for assessing BMI from face images. PLoS One 10, e0140347, doi: 10.1371/journal.pone.0140347 (2015). 26460526 PMC4603950

[b13] Bookstein F. L. Integration, disintegration, and self-similarity: characterizing the scales of shape variation in landmark data. Evol Biol 42, 395–426, doi: 10.1007/s11692-015-9317-8 (2015). 26586921 PMC4642606

[b14] Brown E. & Perrett D. I. What gives a face its gender? Perception 22, 829–840, doi: 10.1068/p220829 (1993). 8115240

[b15] Keating C. F. Gender and the physiognomy of dominance and attractiveness. Soc Psychol Q 48, 61–70, doi: 10.2307/3033782 (1985).

[b16] Gonçalves G. . Once upon a face: the effect of eye size, observer and stimulus gender on impression formation. Curr Psychol 34, 112–120, doi: 10.1007/s12144-014-9244-3 (2015).

[b17] Re D. E. & Rule N. O. The big man has a big mouth: Mouth width correlates with perceived leadership ability and actual leadership performance. J Exp Soc Psychol 63, 86–93, doi: 10.1016/j.jesp.2015.12.005 (2016).

[b18] Rohrich R. J. & Pessa J. E. The fat compartments of the face: Anatomy and clinical implications for cosmetic surgery. Plast Reconstr Surg 119, 2219–2227, doi: 10.1097/01.prs.0000299636.97607.5f (2007). 17519724

[b19] Hess U., Adams R. B. & Kleck R. E. The face is not an empty canvas: how facial expressions interact with facial appearance. Philos Trans R Soc Lond B Biol Sci 364, 3497–3504, doi: 10.1098/rstb.2009.0165 (2009). 19884144 PMC2781893

[b20] Ekman P. About brows: emotional and conversational signals in Human Ethology(eds von Cranach M., Foppa K., Lepenies W. & Ploog D.) 169–248 (Cambridge University Press, 1979).

[b21] Salgado-Montejo A., Salgado C. J., Alvarado J. & Spence C. Simple lines and shapes are associated with, and communicate, distinct emotions. Cogn Emot, 1–15, doi: 10.1080/02699931.2015.1133401 (2016). 26817592

[b22] Palme R. & Möstl E. Measurement of cortisol metabolites in faeces of sheep as a parameter of cortisol concentration in blood. Int J Mammal Biol 62, 192–197 (1997).

[b23] Wallner B., Möstl E., Dittami J. & Prossinger H. Fecal glucocorticoids document stress in female Barbary macaques (*Macaca sylvanus*). Gen Comp Endocrinol 113, 80–86, doi: 10.1006/gcen.1998.7183 (1999). 9882546

[b24] Siart B., Pflüger L. S. & Wallner B. Pulling rank: Military rank affects hormone levels and fairness in an allocation experiment. Front Psychol 7, 1750, doi: 10.3389/fpsyg.2016.01750 (2016). 27891109 PMC5104734

[b25] Windhager S., Schaefer K. & Fink B. Geometric morphometrics of male facial shape in relation to physical strength and perceived attractiveness, dominance, and masculinity. Am J Hum Biol 23, 805–814 (2011). 21957062 10.1002/ajhb.21219

[b26] Bookstein F. L. Measuring and Reasoning: Numerical Inference in the Sciences. (Cambridge University Press, 2014).

[b27] Bookstein F. L. Morphometric Tools for Landmark Data: Geometry and Biology. (Cambridge University Press, 1991).

[b28] Mandelbrot B. B. The Fractal Geometry of Nature. (W. H. Freeman Co., 1982).

[b29] Perrin J. *Les Atomes*. 1913. English edition: *Atoms*, tr. D. Hammick. (Constable, 1923).

[b30] Marcus L. F., Corti M., Loy A., Naylor G. J. P. & Slice D. E. Advances in Morphometrics. (Plenum Press, New York, 1996).

[b31] Rohlf F. J. & Marcus L. F. A revolution in morphometrics. Trends Ecol Evol 8, 129–132 (1993). 21236128 10.1016/0169-5347(93)90024-J

[b32] Bookstein F. L. The inappropriate symmetries of multivariate statistical analysis in geometric morphometrics. Evol Biol 43, 277–313, doi: 10.1007/s11692-016-9382-7 (2016). 27512236 PMC4960290

[b33] Rohlf F. J. The tps series of software. Hystrix 26, 9–12, doi: 10.4404/hystrix-26.1-11264 (2015).

[b34] Grammer K., Fink B., Juette A., Ronzal G. & Thornhill R. Female faces and bodies: n-dimensional feature space and attractiveness in Advances in visual cognition. Volume I: Facial attractiveness(eds Rhodes G. & Zebrowitz L. A.) 91–125 (Ablex Publishing, 2001).

[b35] Sepulveda J. Challenges in routine clinical chemistry testing: analysis of small molecules in Accurate results in the clinical laboratory(eds Dasgupta A. & Sepulveda J. L.) 93–129 (Elsevier, 2013).

[b36] Connell J. M. C. . Effects of ACTH and cortisol administration on blood pressure, electrolyte metabolism, atrial natriuretic peptide and renal function in normal man. J Hypertens 5, 425–433, doi: 10.1097/00004872-198708000-00007 (1987). 2822795

[b37] Folstad I. & Karter A. J. Parasites, bright males, and the immunocompetence handicap. Am Nat 139, 603–622 (1992).

[b38] Coetzee V., Chen J., Perrett D. I. & Stephen I. D. Deciphering faces: quantifiable visual cues to weight. Perception 39, 51–61, doi: 10.1068/p6560 (2010). 20301846

[b39] Wilkinson C. Forensic Facial Reconstruction. (Cambridge University Press, 2004).

[b40] Windhager S., Patocka K. & Schaefer K. Body fat and facial shape are correlated in female adolescents. Am J Hum Biol 25, 847–850, doi: 10.1002/ajhb.22444 (2013). 24105760

[b41] Huang Z., Barrett J. S., Barrett K., Barrett R. & Ng C. M. Novel method to predict body weight in children based on age and morphological facial features. J Clin Pharmacol 55, 447–451, doi: 10.1002/jcph.422 (2015). 25370186

[b42] Henderson A. J., Holzleitner I. J., Talamas S. N. & Perrett D. I. Perception of health from facial cues. Phil Trans R Soc B 371, 20150380, doi: 10.1098/rstb.2015.0380 (2016). 27069057 PMC4843618

[b43] Moore F. R. . Cues to sex- and stress-hormones in the human male face: Functions of glucocorticoids in the immunocompetence handicap hypothesis. Horm Behav 60, 269–274, doi: 10.1016/j.yhbeh.2011.05.010 (2011). 21672543

[b44] Gonzalez-Santoyo I. . The face of female dominance: Women with dominant faces have lower cortisol. Horm Behav 71, 16–21, doi: 10.1016/j.yhbeh.2015.03.006 (2015). 25857930

[b45] Schaal S., Kunsch K. & Kunsch S. Der Mensch in Zahlen. 4th edn (Springer, 2016).

[b46] Feelders R. A., Pulgar S. J., Kempel A. & Pereira A. M. MANAGEMENT OF ENDOCRINE DISEASE: The burden of Cushing’s disease: clinical and health-related quality of life aspects. Eur J Endocrinol 167, 311–326, doi: 10.1530/EJE-11–1095 (2012). 22728347

[b47] Perrett D. In Your Face: The New Science of Human Attraction. (Palgrave MacMillan, 2010).

[b48] Vernon R. J. W., Sutherland C. A. M., Young A. W. & Hartley T. Modeling first impressions from highly variable facial images. Proc Natl Acad Sci USA 111, E3353–3361, doi: 10.1073/pnas.1409860111 (2014). 25071197 PMC4136614

[b49] Blais C., Jack R. E., Scheepers C., Fiset D. & Caldara R. Culture shapes how we look at faces. PLoS One 3, e3022, doi: 10.1371/journal.pone.0003022 (2008). 18714387 PMC2515341

[b50] Tan C. B. Y., Sheppard E. & Stephen I. D. A change in strategy: Static emotion recognition in Malaysian Chinese. Cogent Psychol 2, 1085941, doi: 10.1080/23311908.2015.1085941 (2015).

